# The Evaluation of Cognitive Impairment on Dental Care Utilization: Comparative Analysis of Insurance Types

**DOI:** 10.1093/geroni/igaf122.1225

**Published:** 2025-12-31

**Authors:** Zhang Zhang, Bei Wu

**Affiliations:** Johns Hopkins Bloomberg School of Public Health, Baltimore, Maryland, United States; NYU Shanghai, Shanghai, China (People’s Republic)

## Abstract

**Background:**

Dental care is critical for overall health and quality of life, yet barriers to access persist, particularly for individuals with disabilities. This study examines the relationship between cognitive impairment and dental care utilization, focusing on differential effects across insurance types.

**Method:**

Using the Health and Retirement Study (HRS) from 1996 to 2018, we constructed a longitudinal sample cohort of 25,201 participants aged at or above 65 with 127,217 observations. We adopted an individual fixed-effect model to measure the relationship between cognitive impairment and the probability of using dental care in the past two years and examined the heterogeneous effect across insurance types. We defined cognitive impairment using the Langa–Weir classification method.

**Results:**

4.4 % have no insurance, 38.4% are in Medicare only, 9.8% are dual-eligible in Medicare and Medicaid, and 47.4% are in Medicare and private insurance. Compared with the healthy group, cognitive impairment was significantly associated with reducing the probability of dental care utilization by 7.6 percentage points (p < 0.01, 95% CI: -0.084, -0.068). Moreover, cognitive impairment was significantly associated with declining dental visits by 7.7 percentage points (p < 0.01, 95% CI: -0.151, -0.003) among no insurance and by 1.9 percentage points (p < 0.01, 95% CI: -0.035, -0.003) among Medicare-only, but no significant association among dual eligibles or those with private insurance.

**Conclusion:**

This study reveals the relationship between cognitive impairment and reduction in dental care utilization among heterogeneous insurance groups, particularly among non-insurance and Medicare-only. These findings underscore the urgent need for Medicare reforms to include dental coverage.

